# Supplementary material for: The causal relationship between the human gut microbiota and pyogenic arthritis: a Mendelian randomization study
Source: Front Cell Infect Microbiol. 2024 Nov 26;14:1452480. doi: 10.3389/fcimb.2024.1452480 (PMC11629706; doi:10.3389/fcimb.2024.1452480)
Supplement: Supplementary file 1 [file DataSheet1.zip › mendelian test/ebi-a-GCST90027540.csv_leave_one_out.pdf]

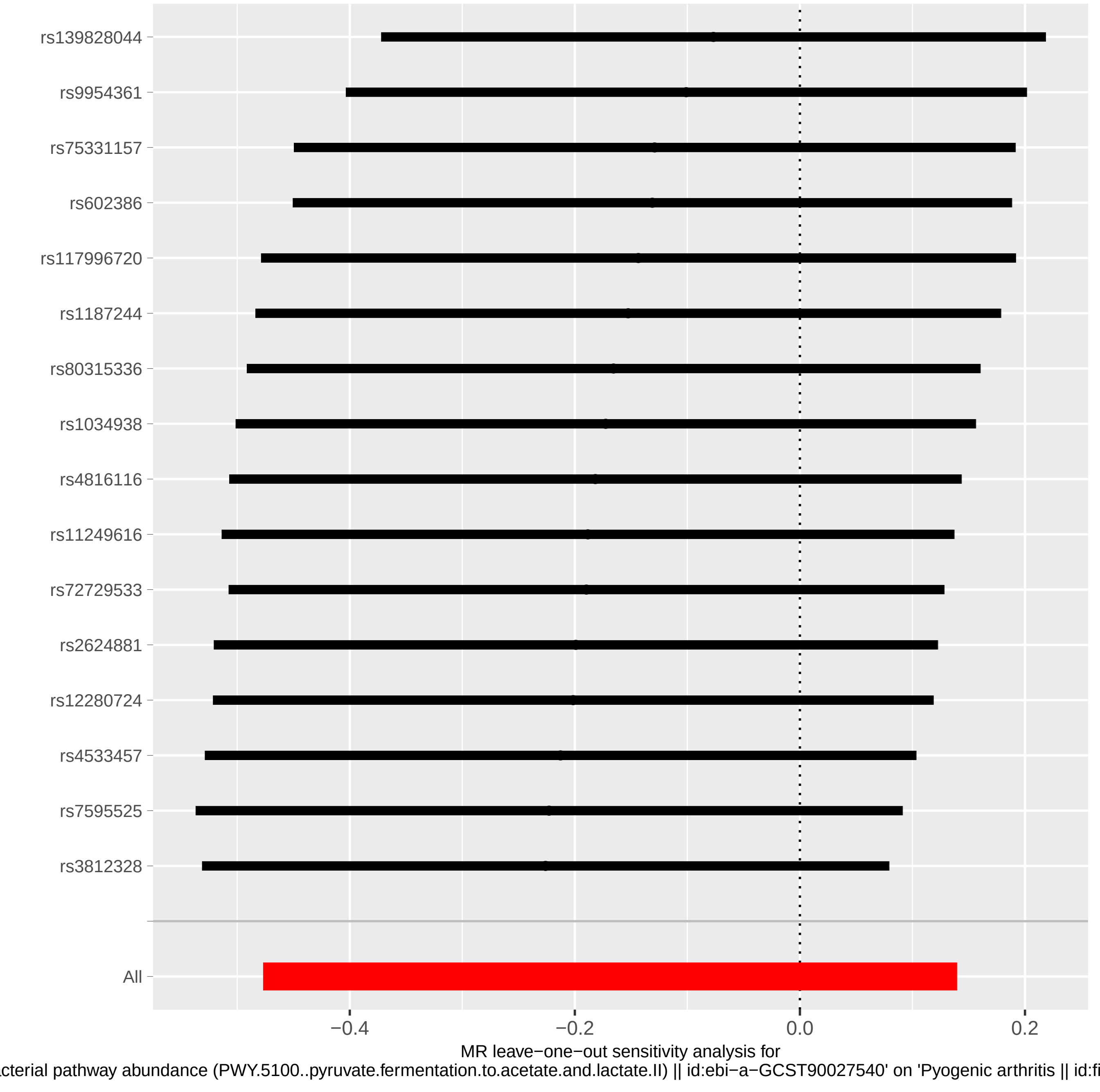
rs139828044
rs9954361
rs75331157
rs602386
rs117996720
rs1187244
rs80315336
rs1034938
rs4816116
rs11249616
rs72729533
rs2624881
rs12280724
rs4533457
rs7595525
rs3812328
All
−0.4
−0.2
0.0
0.2
MR leave−one−out sensitivity analysis for
cterial pathway abundance (PWY.5100..pyruvate.fermentation.to.acetate.and.lactate.II) || id:ebi−a−GCST90027540' on 'Pyogenic arthritis || id:fi
